# Of the Influence of Cold upon the Health of the Inhabitants of London

**Published:** 1800

**Authors:** William Heberden


XXI. *Of the Influence of Cold upon the Health of the Inhabitants of London. By* William Heberden, *Jun. M. D. F. R. S.* Vide *Philoſophical Tranſactions of the Royal Society of London, for the Year* 1796. *Part II.* 4to. London, 1796.

THE extraordinary mildneſs of January, 1796, compared with the unuſual ſeverity of the ſame month of the preceding year, ſeemed, to the author of the paper before us, to afford a peculiarly favourable opportunity of obſerving the effect of each of theſe ſeaſons contraſted contrafted with each other. For of thefe two fucceffive winters, he obferves, one was the coldeft and the other the warmeft, of which any regular account has ever been kept in this country.

During January, 1796, he remarks, nothing was more common than to hear expreffions of the unfeafonablenefs of the weather; and fears left the want of the ufual degree of cold, should be productive of difeafe: "a bracing "cold and a clear froft," being familiar in the mouth of every Englifhman; and what he is taught to wifh for, as among the greateft promoters of health and vigour. But whatever deference may be due to received opinions, Dr. Heberden certainly makes it appear, from the moft fatisfactory evidence, that the prejudices of the world are upon this point at leaft unfounded.

The average degrees of heat, he obferves, upon Fahrenheit's thermometer kept in London during the month of January 1795, was 23° in the morning, and 29°.4 in the afternoon. The average in January 1796, was 43°.5 in the morning, and 50°.1 in the afternoon. A difference above twenty degrees. And if we turn our attention from the comparative coldneſs of theſe months, to the correſponding healthineſs of each, collected from the weekly bills of mortality, we ſhall find, he obſerves, the reſult no leſs remarkable. For in five weeks between the 31ſt of December 1794 and the 3d of February 1795, the whole number of burials, it ſeems, amounted to 2823; and in an equal period of five weeks between the 30th of December 1795 and the 2d of February 1796, to 1471. So that the exceſs of the mortality in January 1795 above that of January 1796, was not leſs than of 1352 perſons. And though he has only given the evidence of two years, the ſame concluſion, he aſſures us, may univerſally be drawn; as he has learned from a careful examination of the weekly bills of mortality for many years.

After ſtating theſe facts, Dr. Heberden proceeds to conſider a little more particularly the ſeveral ways in which increaſe of mortality may be ſuppoſed to be produced; and to point out ſome of the principal injuries which people are liable to ſuſtain in their health from a ſevere froſt. And one of the firſt things, he remarks, and that muſt ſtrike every mind engaged in this inveſtigation, is its effect on old people. It is curious, ſays he, to obſerve among thoſe who are are ſaid in the bills to die above 60 years of age, how regularly the tide of mortality follows the influence of this prevailing cauſe: ſo that a perſon uſed to ſuch enquiries, may, he thinks, form no contemptible judgment of the ſeverity of any of our winter months, merely by attending to this circumſtance. Thus he finds that their number in January, 1796, was not much above ⅕th of what it had been in the ſame month the year before. The article of aſthma, he obſerves, is, as might be expected, prodigiouſly increaſed, and perhaps includes no inconſiderable part of the mortality of the aged. After theſe come apoplexies and palſies, fevers, conſumptions, and dropſies. Under the two laſt of which, he preſumes, are contained a large proportion of the chronical diſeaſes of this country, all which ſeem to be hurried on to a premature termination.

Dr. Heberden gives the reſults of his enquiry in the following table:—

### 1795

| Week ending | Mean heat | | Whole No. of deaths | Aged above 60. | Aſthma | Apo-plexy and palſy | Fever | Con-ſump-tion | Dropſy |
|---|---|---|---|---|---|---|---|---|---|
| | Morn. | Noon. | | | | | | | |
| 6 Jan. | 25° | 29° | 244 | 51 | 13 | 4 | 20 | 73 | 7 |
| 13 Jan. | 26° | 32° | 532 | 139 | 26 | 13 | 49 | 158 | 20 |
| 20 Jan. | 24° | 30° | 637 | 145 | 51 | 11 | 81 | 164 | 37 |
| 27 Jan. | 19° | 27° | 543 | 143 | 64 | 11 | 42 | 157 | 17 |
| 3 Feb. | 25° | 37° | 867 | 239 | 95 | 13 | 66 | 273 | 45 |
| Reſult | 23° | 29°.4 | 2823 | 717 | 249 | 52 | 258 | 825 | 126 |

### 1796

| Week ending | Mean heat | | Whole No. of deaths | Aged above 60 | Aſthma | Apo-plexy and palſy | Fever | Con-ſump-tion | Dropſy |
|---|---|---|---|---|---|---|---|---|---|
| | Morn. | Noon. | | | | | | | |
| 5 Jan. | 40° | 46° | 300 | 35 | 5 | 7 | 34 | 79 | 13 |
| 12 Jan. | 41° | 49° | 273 | 37 | 9 | 5 | 25 | 53 | 19 |
| 19 Jan. | 48° | 53° | 313 | 29 | 2 | 4 | 29 | 77 | 11 |
| 26 Jan. | 47° | 52° | 257 | 20 | 7 | 9 | 23 | 47 | 11 |
| 2 Feb. | 41° | 49° | 328 | 32 | 6 | 6 | 23 | 86 | 16 |
| Reſult | 43°.5 | 50°.1 | 1471 | 153 | 29 | 31 | 134 | 342 | 70 |

Dr.

Dr. Heberden obferves, that notwithftanding the plague, the remittent fever, the dyfentery, and the fcurvy, have fo decreafed, that their very name is almoft unknown in London; yet there has arifen a prejudice concerning putrid difeafes, which feems to have made people more and more apprehenfive of them, as the danger has been growing lefs. It muft in great meafure, he thinks, be attributed to this, that the confumption of Peruvian bark in this country has, within the laft fifty years, increafed from 14,000 to above 100,000 pounds annually. And the fame caufe, he imagines, has probably contributed, from a miftaken mode of reafoning, to prepoffefs people with the idea of the wholefomenefs of a hard froft. But it has in another place,* he obferves, been demonftrated, that a long froft is eventually productive of the worft putrid fevers that are at this time known in London; and that heat does in fact prove a real preventive againft that difeafe. And although this may be faid to be a very remote effect of the cold, it is not therefore, he

* Obfervations on the Jail Fever, by Dr. Hunter, Med. Trans. Vol. III.

thinks, the leſs real in its influence upon the mortality of London. Accordingly a compariſon of the numbers in the foregoing table, he obſerves, will ſhow that very nearly twice as many perſons died of fevers in January 1795, as did in the correſponding month of 1796. He has found too, that in the winter of 1795, the true ſcurvy was generated in the metropolis from the ſame cauſes extended to an unuſual length. But theſe, he remarks, are by no means the only, nor indeed, in his opinion, the principal ways, in which a froſt operates to the deſtruction of great numbers of people. The poor as they are worſe protected from the weather, ſo are they of courſe the greateſt ſufferers by its inclemency. But every phyſician, he obſerves, in London, and every apothecary, can add his teſtimony, that their buſineſs among all ranks of people never fails to increaſe, and to decreaſe with the froſt.

Dr. Heberden points out the dangerous and fatal effects which may be expected to ariſe from an imprudent expoſure to cold, in a country like this, where the prevailing complaints among all orders of people, are colds, coughs, conſumptions and rheumatiſms; and concludes his paper with obſerving, that many many doctrines, very importantly erroneous, are daily impofed upon the world for want of attention to this great truth; that it is from general effects only, and thofe founded upon extenfive experience, that any maxim to which each individual may with confidence defer, can poffibly be eftablifhed.

